# Skin and soft tissue infections in the intensive care unit: a retrospective study in a tertiary care center

**DOI:** 10.5935/0103-507X.20170019

**Published:** 2017

**Authors:** Luís Filipe Malheiro, Rita Magano, Alcina Ferreira, António Sarmento, Lurdes Santos

**Affiliations:** 1 Serviço de Moléstias Infecciosas, Centro Hospitalar de São João - Porto, Portugal.; 2 Grupo de Pesquisa e Desenvolvimento em Nefrologia e Moléstias Infecciosas, Instituto de Engenharia Biomédica, Universidade do Porto - Porto, Portugal.; 3 Serviço de Moléstias Infecciosas, Centro Hospitalar e Universitário de Coimbra - Coimbra, Portugal.

**Keywords:** Skin manifestations, Abscess, Cellulitis, Fasciitis, necrotizing, Sepsis, Risk factors, Intensive care units

## Abstract

**Objective:**

To identify factors that may influence outcomes in patients with severe skin and soft tissue infections in the intensive care unit.

**Methods:**

A retrospective observational study was conducted in a cohort of 1,123 critically ill patients admitted to an intensive care unit with a primary or secondary diagnosis of severe skin and soft tissues infection between January 2006 and December 2014.

**Results:**

Thirty patients were included, 20 (66.7%) of whom were diagnosed with necrotizing fasciitis; in these patients, perineal area involvement was most commonly identified. Abscess was diagnosed in 8 (26.7%) patients, most commonly involving the cervical area. Risk factors such as immunosuppression and previous surgical trauma were commonly observed in this population. The most commonly isolated microorganism was *Escherichia coli*. Multidrug resistant microorganisms were commonly detected, even in the absence of traditional risk factors; among these patients, previous use of antibiotics was the most common risk factor for drug resistance. The rate of mortality was significantly higher in patients with necrotizing fasciitis (55%, p = 0.035) and associated with disease severity, presence of septic shock, cardiac arrest and leucocytosis.

**Conclusion:**

Different risk factors and etiologies of severe skin and soft tissue infections were identified. Necrotizing fasciitis and drug-resistant bacteria were significant predictors of mortality, even in the absence of traditional risk factors. Obtaining a better understanding of trends in the risk factors and microorganisms associated with severe skin infections may help in the determination of prompt treatment and antibiotic choices.

## Abstract (pt)

**Objetivo:**

Descrever o prognóstico, os fatores de risco e a etiologia das infecções da pele e dos tecidos moles na unidade de terapia intensiva.

**Métodos:**

Estudo retrospectivo de uma coorte de 1.123 pacientes graves admitidos a uma unidade de terapia intensiva com o diagnóstico de infecção grave de pele ou tecidos moles.

**Resultados:**

Foram selecionados 30 pacientes, sendo 20 (66,7%) com fasceíte necrotizante, predominantemente da região perineal; 8 (26,7%) com abscesso cutâneo; e 2 (6,6%) com celulite. A maioria dos pacientes tinha fatores de risco, como imunossupressão e lesões cutâneas. O microrganismo isolado predominante foi *Escherichia coli*. Pacientes com fasceíte necrotizante na admissão à unidade de terapia intensiva apresentaram mortalidade significativamente maior (55%; p = 0,035), assim como aqueles com maior índice de severidade, choque séptico, parada cardiorrespiratória e leucocitose. Organismos resistentes à antibioticoterapia foram comuns, mesmo na ausência de fatores de risco. Quando presente, o fator de risco mais comum foi o uso prévio de antibiótico.

**Conclusão:**

Foram identificados fatores de risco e microrganismos diferentes dos classicamente descritos na literatura, além de elevada mortalidade da fasceíte necrotizante e presença de microrganismos multirresistentes na ausência de fatores de risco. Dada a aparente evolução etiológica das infecções da pele e tecidos moles, a identificação de novos fatores de risco e etiologia pode contribuir para uma terapêutica antimicrobiana mais adequada.

## INTRODUCTION

Skin and soft tissue infections (SSTI) are among the most common conditions observed in patients presenting to emergency departments; these infections are sometimes severe enough to induce septic shock and justify intensive care unit (ICU) admission.^(1,2)^ Severe SSTI have been found to be responsible for 4.3 - 10.5% of septic episodes and have a case fatality rate that varies between 1.3 - 7.2%.^(3,4)^ Aggressive fluid resuscitation, intravenous antibiotics, surgical procedures and supportive care are generally required in the treatment of severe SSTI.^(5)^ Surgical debridement has been identified as a major determinant of outcomes in these patients, as septic shock will not resolve until all the infected/necrotic tissue has been removed and local infection control has been achieved.^(6,7)^ Due to the high rates of mortality and morbidity associated with SSTI, knowledge of the etiology of and risk factors for these infections is crucial in the effort to provide quality healthcare. In this study, we aimed to assess and describe the epidemiology and etiology of severe SSTI and identify factors associated with outcomes in severely infected ICU patients.

## METHODS

The study was a retrospective observational study that included a cohort of critically ill patients admitted in the ICU of the Infectious Diseases Department (ICU-ID) in an urban teaching hospital between January of 2006 and May of 2014. The inclusion criterion was a primary or secondary diagnosis of severe skin and soft tissues infection (necrotizing fasciitis, abscess or cellulitis). The exclusion criteria were the absence of clinical information or loss to follow-up after discharge from the ICU-ID (i.e., transfer to another institution). Intra-institutional Ethics Committee approval was obtained, and all included patients provided written informed consent. Confidentiality was maintained through name and medical record deidentification. The records of 30 patients out of the total 1,123 patients admitted in the ICU-ID were reviewed. The included patients had not been lost to follow-up, and all relevant clinical data were available for these patients. Sociodemographic, blood analysis, Simplified Acute Physiology severity score (SAPS II),^(8)^ therapeutic procedure and outcome (length of stay in the ICU, mortality in the ICU and mortality after 28-days) data were collected. Necrotizing fasciitis (NF) was further classified in terms of microbiological characteristics as type I (polymicrobial), type II (monomicrobial) and type III (*Clostridial* or rarer agents).^(2)^

For the statistical analysis, means or medians with standard deviations (SD), interquartile ranges (IQR), minimums and maximums are reported for continuous variables, and proportions are reported for categorical variables. Categorical variables were compared using either the chi-square or exact Fisher tests as appropriate; additionally, relative risks (RR) with 95% confidence intervals (CI95%) were calculated. The means of continuous variables were compared using either *t*-tests or Mann-Whitney U tests, as appropriate. The analyses were performed using Statistical Package for Social Sciences (SPSS) version 22.0.0 software.

## RESULTS

A total of 30 patients admitted to the ICU-ID (2.6% of the total number of patients) were diagnosed with severe SSTIs, with a 1:1 male:female ratio and a mean age of 58 years (SD 16.8) identified (Table 1). Thirteen patients (43.3%) were directly admitted to the ICU, and 17 (56.7%) patients were transferred from medical or surgical wards.

**Table 1:** Description of the demographics, risk factors, microbiology, treatment and outcomes

	Necrotizing fasciitis (N = 20)	Abscess (N = 8)	Cellulitis (N = 2)
Demographics			
Age (years)	56.5 (IQR 22)	66.0 (IQR 33)	60 (55, 65)
Sex Male: Female (%/%)	10:10 (50%/50%)	5:3 (62.5:37.5%)	0:2 (100% female)
Microbiology and treatment			
Infection location and classification	Fournier’s gangrene (n = 9):	Cervical/thoracic (n = 5)	Abdominal (n = 1)
	Type I (n = 4)		
	Type II (n = 3)	Other locations (n = 3):	Lower limb (n = 1)
	No MO identified (n = 2)	Abdominal wall	
	Cervical/thoracic fasciitis (n = 6):	Lumbar area	
	Type I (n = 1)	Lower limb	
	Type II (n = 2)		
	No MO identified (n = 3)		
	Abdominal wall/limbs fasciitis (n = 5):		
	Type II (n = 3)		
	No MO identified (n = 2)		
Isolated microorganisms	Fournier’s gangrene (n = 9):	*Staphylococcus aureus* (n = 2)	*Staphylococcus hemolyticus* (n = 1)
	*Enterobacteriacea*	*Mycobacterium tuberculosis* (n = 1)	*Escherichia coli* (n = 1)
	*Escherichia coli* (n = 5)	*Streptococcus mitis* (n = 1)	
	*Enterobacter aerogenes* (n = 1)	*Staphylococcus saprophyticus* (n = 1)	
	*Proteus mirabilis* (n = 1)	*Streptococcus anginosus* (n = 1)	
	*Streptococcus sanguis/gordonii* (n = 2)		
	*Staphylococcus aureus* (n = 1)		
	Cervical/thoracic fasciitis (n = 6):		
	*Acinetobacter baumanii* (n = 1)		
	*Klebsiella pneumoniae* (n = 1)		
	*Enterococcus faecalis* (n = 1)		
	*Staphylococcus aureus* (n = 1)		
	Abdominal wall/limbs fasciitis (n = 5):		
	*Escherichia coli* (n = 1)		
	*Klebsiella pneumoniae* (n = 1)		
	*Enterobacter cloacae* (n = 1)		
Multidrug resistance	MDR - 5/20 (25%) patients	MDR - 1/8 (12.5%) patients	MDR - 1/2 (50%) patients
Risk factors for multidrug resistance	Previous antibiotic use - 6/20 (30%) patients	Previous antibiotic use - 2/8 (25%) patients	Previous antibiotic use - 1/2 (50%) patients
	Previous contact with healthcare or hospital admission > 48 hours before symptom initiation - 5/20 (25%) patients	Previous contact with healthcare or hospital admission > 48 hours before symptom initiation - 1/8 (12.5%) patients	
Sample positivity rates	Pus/drainage: 12/12 positive samples	Pus/drainage: 5/6 positive samples	Pus/drainage: not collected
	Wound swab: 2/7 positive samples	Wound swab: 0/4 positive samples	Wound swab: not collected
	Blood cultures: 4/19 positive samples	Blood cultures: 2/6 positive samples	Blood cultures: 2/2 positive samples
Surgical treatment	Number of patients submitted to procedure:	Number of patients submitted to procedure:	Number of patients submitted to procedure:
	Percutaneous drainage: 7/20 (35%)	Percutaneous drainage: 1/8 (12.5%)	Surgical procedure: 1/2 (50%)
	Surgical debridement: 20/20 (100%)	Surgical drainage: 4/8 (50%)	
	Median number of days between diagnose and surgery: 0 (IQR 2)	Median number of days between diagnose and drainage: 0.5 (IQR 2)	Median number of days between diagnose and surgery: 15
	Median number of surgical procedures: 1 (IQR 2) per patient	Median number of surgical procedures: 2.5 (IQR 3) per patient	
Adjunctive therapies	Hyperbaric oxygen therapy: 2/20 (10%) patients	-	-
	Negative-pressure wound therapy: 2/20 (10%) patients		
Characteristic and severity score upon admission			
Leucocytes (/mm^3^)	12.920 (IQR 16.000)	12.610 (IQR 11.000)	9.540 (7-000, 12.000)
C-reactive protein (mg/L)	192 (IQR 171)	249 (IQR 333)	54 (7 - 102)
Septic shock upon ICU admission	13/20 (65%)	6/8 (75%)	2/2 (100%)
Cardiac arrest during infection	3/20 (15%)	2/8 (25%)	-
SAPS II score	49 (IQR 25) points	44 (IQR 14) points	65 (45 - 85) points

IQR - interquartile ranges; MO - microorganism; MDR - multidrug resistant; ICU - intensive care unit; SAPS II - Simplified Acute Physiology Score.

The identified risk factors (RF) are presented in table 2. Necrotizing fasciitis was diagnosed in 20 (66.7%) patients, with perineal region (Fournier's gangrene) involvement most frequently identified (9; 30%] patients).

**Table 2:** Description of the risk factors

Necrotizing fasciitis (N = 20)	Abscess (N = 8)	Cellulitis (N = 2)
Any immunosuppression (n = 11)	Previous cutaneous or soft tissue infection (n = 5)	Previous surgery (n = 1)
Previous cutaneous or soft tissue infection (n = 9)	Any immunosuppression (n = 4)	Previous cutaneous infection (n = 1)
Type 2 diabetes mellitus (n = 7)	Type 2 diabetes mellitus (n = 2)	Type 2 diabetes mellitus (n = 1)
Previous surgery (n = 4)	Cirrhosis (n = 1)	Cirrhosis (n = 1)
Immunosuppressive drug use (n = 2)	HIV (n = 1)	Malignancy (n = 1)
HIV (n = 2)	Intravenous drug abuse (n = 1)	
Obesity (n = 2)	Immunosuppressive drug use (n = 1)	
Chronic renal failure (n = 1)	Previous trauma (n = 1)	
Malignancy (n = 1)		
Previous surgery (n = 1)		
Tracheostomy (n = 1)		
Intravenous drug abuse (n = 1)		

Abscess was diagnosed in 8 (26.7%) patients, most commonly identified in the cervical region (3; 10%] patients). Cellulitis was diagnosed in 2 (6.7%) patients, affecting the abdominal region and the lower limb region in 1 patient each.

Twenty-one (70%) patients had community-acquired infections, while 9 (30%) patients had healthcare-associated infections. Risk factors for severe cutaneous infections were identified in 18 (58.1%) patients. Three RFs were ascertained in 3 (9.7%) patients, while 2 RFs were ascertained in 5 (16.1%) patients, and only 1 RF was ascertained in 10 (32.3%) patients.

Positive microbiological results were detected in samples obtained by surgical drainage, wound swab or blood culture in 21 (70%) patients (Table 1). The positivity rate was highest in surgical drainage specimens, followed by blood culture specimens. Type I infections were most commonly associated with the perineal area than any other location (RR 3.4; 95%CI 0.79 - 4.7; p = 0.049).

Multidrug resistant (MDR) bacteria were isolated from 7 (23.3%) patients. The presence of MDR bacteria was not significantly associated with any of the identified risk factors, infection type, infection location, or length of overall or ICU hospitalization. MDR isolation was significantly associated with previous use of any antibiotics (RR 4.0, 95%CI 0.85 - 9.50, p = 0.049) and marginally associated with previous contact with healthcare facilities (RR 2.88, 95%CI 0.716 - 7.436, p = 0.067).

**Figure 1:**
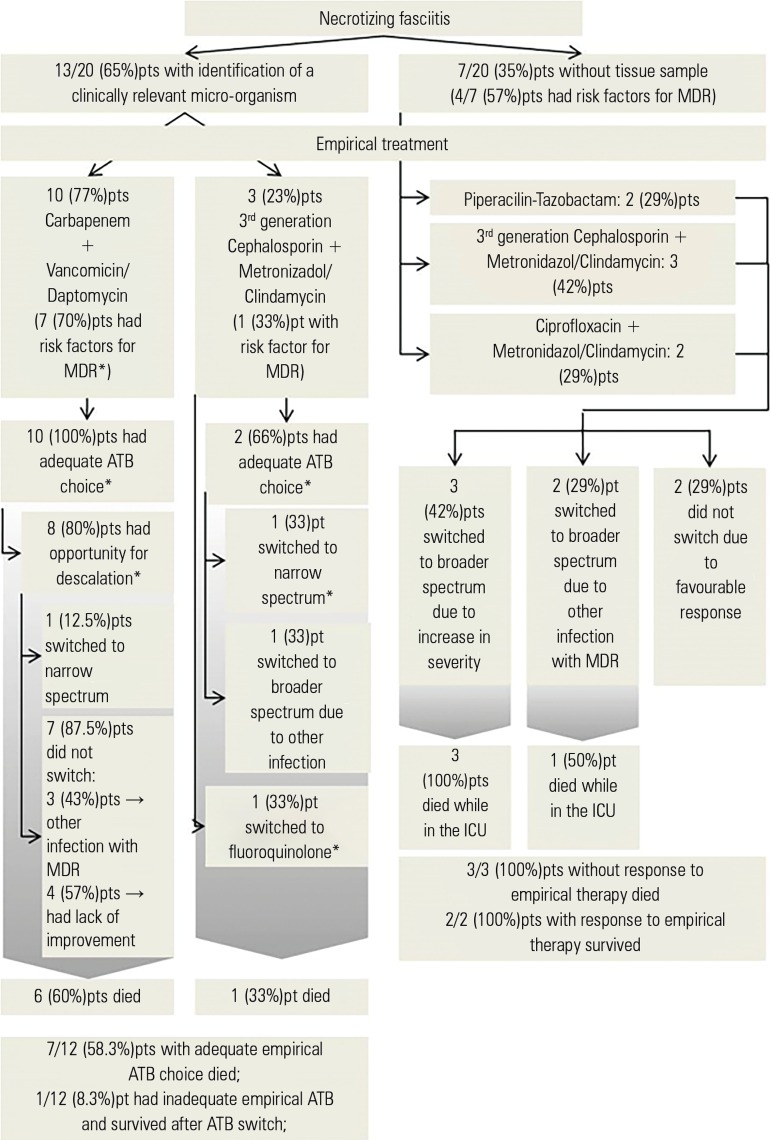
Empirical antimicrobial treatment selected for necrotizing fasciitis. MDR - multi drug-resistant; Pt/Pts - patient/patients; ATB - antibiotic; ICU - intensive care unit. * As determined by antibiotic sensitivity testing.

**Figure 2:**
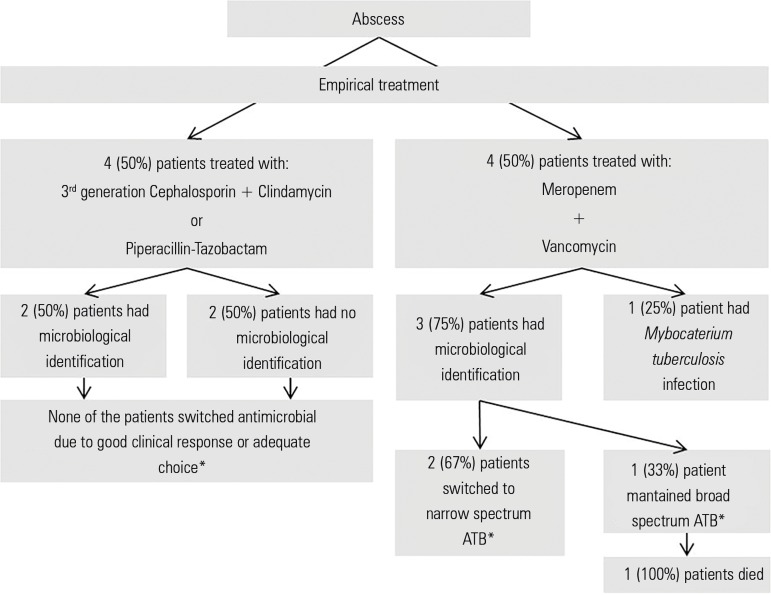
Empirical antimicrobial treatment selected for abscesses. ATB - antibiotic. * as determined by antibiotic sensitivity testing.

**Figure 3:**
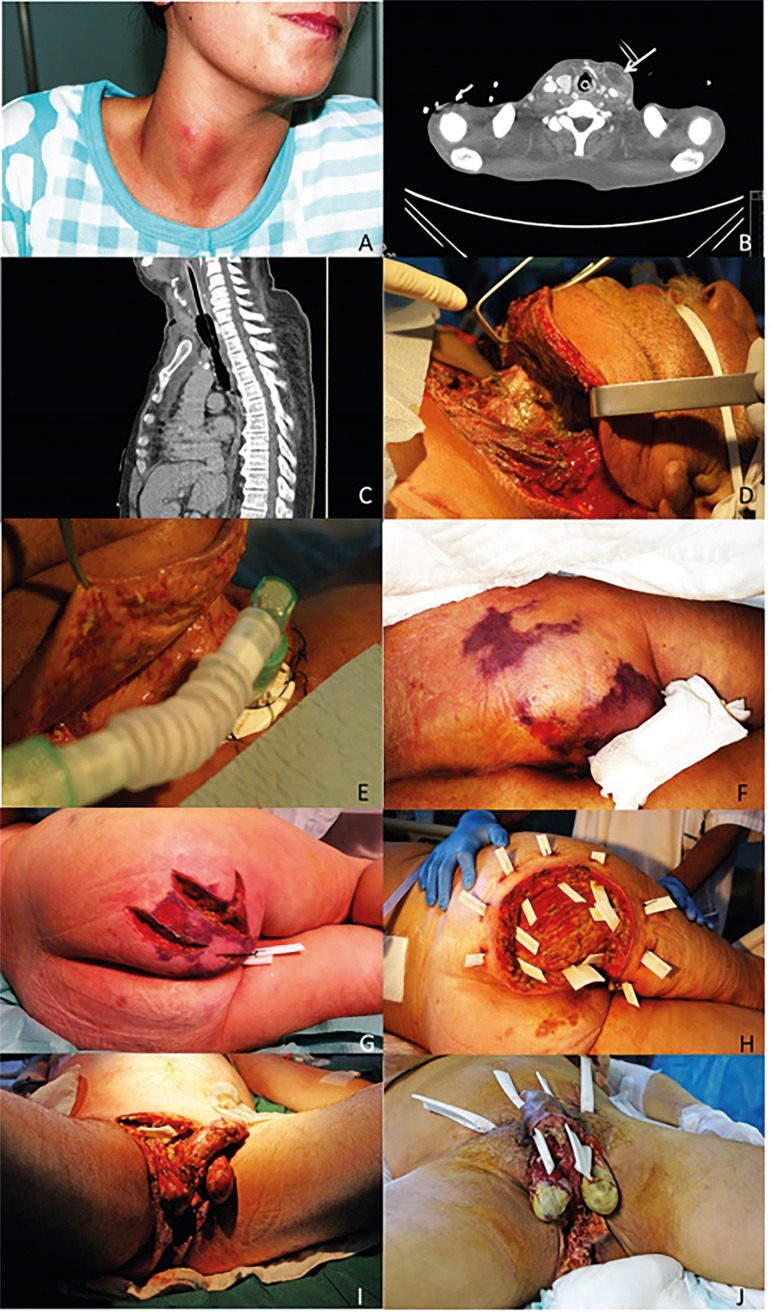
(A) Female patient presenting with cervical abscess prior to surgical drainage. (B) Cervical computed tomography scan revealing a left paratracheal abscess (arrow) in a patient with odontogenic abscess. (C) Thoracic computed tomography scan revealing multiple infectious lesions in the anterior mediastinum that coalesce into a cervical abscess. (D) Surgical debridement of a male patient with cervical necrotizing fasciitis, revealing devitalized muscle and pus in the deep cervical spaces. The patient later underwent surgical tracheostomy (E). (F) Female patient presenting with septic shock and inflammatory signs in the right buttock that extended to the pelvis and vulva. The patient underwent several surgical procedures starting with tissue debridement (G) and extensive devitalized tissue removal and placement of subcutaneous drains (H). (I) Male patient with Fournier's gangrene who underwent extensive perineal debridement. J) Another patient with Fournier's gangrene and several surgical drains in place.

Data on empirical antimicrobial treatment are located in figure 1 and figure 2. Data on surgical treatment is presented in table 1. Surgical wound debridement was performed on 27 (90%) patients, with a median of 1 procedure and a range of 1 - 4 procedures performed. The surgical approaches utilized included extensive debridement and drainage (Figure 3). Four patients with NF required adjunctive treatments such as negative-pressure wound therapy and hyperbaric oxygen therapy to encourage favorable wound evolution.

Empirical antibiotic treatment was provided to every patient and adapted whenever possible to the clinical evolution and microbiological characteristics of the patient. All NF patients who had a sample collected and a microorganism isolated received broad spectrum antibiotics with or without MDR coverage. In a high proportion (80%) of these patients, there was an opportunity to switch to a narrower spectrum antibiotic (de-escalation). However, due to lack of improvement (4 of 13; 30.8% patients) or the presence of other infections (4 of 13; 30.8% patients), de-escalation was only possible in 2 of the 13 (15.4%) patients on whom antimicrobial sensitivity tests had been performed. Only one (7.7%) patient received inadequate empirical antibiotic therapy and survived.

Data on the outcomes of each infection can be found in table 3. In-hospital mortality was identified in 14 (46.7%) patients; of these patients, 12 (40.0%) died while in the ICU and 2 (6.7%) died after discharge from the ICU, of whom 1 (3.3%) died more than 28 days after ICU admission due to NF.

**Table 3:** Description of the outcomes of patients with skin and soft tissue infection

	Necrotizing fasciitis (N = 20)	Abscess (N = 8)	Cellulitis (N = 2)
Length of stay in ICU (days)	8 (IQR 14)	14 (IQR 43)	21 (10, 31)
Length of stay in hospital (days)	53 (IQR 45)	27 (IQR 51)	60.5 (20, 101)
Need for mechanical ventilation	17/20 (85%)	8/8 (100%)	2/2 (100%)
Need for renal rep. therapy (prevalence and duration in days)	4/20 (20%)	2/8 (20%)	-
	14.5 (IQR 19) days	6 (0, 14) days	
Other nosocomial infection in the ICU	8/20 (40%)	1/8 (12.5%)	-
Overall mortality	11/20 (55%)	1/8 (12.5%)	2/2 (100%)
ICU mortality	10/20 (50%)	1/8 (12.5%)	1/2 (50%)
Mortality in the first 48 hours	4/20 (20%)	-	1/2 (50%)
Mortality within 28 days	6/20 (50%)	1/8 (12.5%)	1/2 (50%)
Mortality rate by infection location	Fournier’s gangrene: 6/9 (66.6%)	The patient that died had a lower back abscess develop in	Both patients died
	Cervical: 1/5 (20%)	the context of a disseminated *S. aureus *infection	
	Thorax: 1/1 (100%)		
	Abdominal wall: 1/2 (50%)		
	Limbs: 2/3 (66.6%)		
Outcomes following hospital discharge	3/9 (33%) patients with Fournier’s gangrene needed extensive plastic surgery, and 2/9 (22%) of these patients required hyperbaric oxygen therapy;	7/10 (70%) patients survived and demonstrated adequate wound healing	None survived
	1/3 (33%) patient with limb fasciitis underwent limb amputation;		
	4/6 (67%) patients with cervical fasciitis recovered with adequate wound healing;		
	1/2 (50%) patient with abdominal wall fasciitis required negative-pressure wound therapy		

ICU - intensive care unit; IQR - interquartile ranges.

The risk of mortality was significantly higher in patients with NF than cutaneous abscess patients (RR 4.4; 95%CI 0.67 - 28.70; p = 0.035).

A total amount of 5 patients (16.7%) suffered cardiac arrest. These events were not directly responsible for the death of any patient, but when present, cardiac arrest was significantly associated with the risk of mortality in the ICU (p = 0.046; RR 3.4, 95%CI 0.57 - 20.01).

Septic shock (RR 2.4; 95%CI 0.90 - 5.94; p = 0.041) and median leucocytes count (18.540/mm^3^ *versus* 10.810/mm^3^; p = 0.011) were also significantly associated with the risk of mortality in the ICU.

Mortality was not significantly associated with any other variable including age, gender, infection type (type 1 *versus* type 2) or location, duration between admission and surgery, empirical antibiotic adequacy, risk factors or isolation of MDR bacteria.

## DISCUSSION

In this study, we highlighted the high rates of SSTI morbidity and mortality and further characterized the risk factors, etiological patterns and treatment choices associated with SSTIs. An overall incidence of SSTI of 2.6% was observed in the total patient population admitted to the ICU over the course of 6 years. The annual incidence rate was 2.9 SSTIs per 100 admissions.

### Infection classification and risk factors

The perineal region was the most common location in which NF was observed in our study, and no gender predominance was identified, findings that are in contrast with the results of previous studies reporting the infection to be more common in males and in the limbs.^(2,9-11)^ This difference may be related to the presence of other conditions, such as previous surgical procedures and untreated perineal infections, and the fact that patients in this study resided in urban areas and were less likely to experience daily trauma due to engaging in manual labor.^(12)^

Cervical NF almost invariably occurred secondary to an oral or cervical infection. Mediastinitis is a complication that may occur in some cervical fasciitis cases when the infection accesses the superior thoracic region through the retropharyngeal space. This infection may be clinically difficult to detect in its initial phase and, in our study, was generally only diagnosed after the performance of initial debridement and upon re-evaluation via computed tomography scan (CT-scan).

Half of the patients had no identifiable cause of immunodeficiency, suggesting that healthy patients may also develop SSTIs, as observed in other studies.^(13)^

Abscesses and cellulitis, classically considered uncomplicated infections, may become severe if they involve life-threatening anatomical sites, such as the perineal or cervical areas, or are accompanied by sepsis.^(5,14)^ The main complications identified in abscess patients in our study were progression of the infection to the mediastinum and airway obstruction, and some cases were complicated by septic shock. Immunosuppression (either drug-induced or secondary to HIV infection or cancer) was commonly seen in patients with NF, abscess and cellulitis.

### Etiology and diagnosis

Our study revealed *Escherichia coli* to be the most commonly isolated pathogen in NF, even in patients with community-acquired infections; this finding is in contrast with previous studies identifying *Staphylococcus aureus* and β-hemolytic *Streptococci* (groups A, C and G) as the most common microorganisms.^(5,15-17)^ Factors that we believe may have contributed to this finding include the high prevalence of risk factors related to contact with healthcare, surgical iatrogeny and immunosuppression. This finding may also reflect the predominance of perineal involvement, which was likely identified due to the proximity of this region to the genitourinary and gastrointestinal tracts and the fact that many of these cases occurred secondary to perianal or perineal abscesses, in which Gram-negative bacteria are more commonly involved.^(18,19)^

Abscesses were most commonly associated with oral *Streptococcus* species, which was reflected by the high number of cervical/thoracic infections. Although surgical drainage specimens were submitted for the majority of the abscess patients, anaerobic microorganisms were not isolated from any of the abscesses. In patients with HIV and a solitary abscess, infection with *Mycobacterium tuberculosis* should also be considered.

An important conclusion that may be derived from this study is that microbiological samples should always be collected, especially during surgical debridement or abscess drainage. Similar to the results of other studies, a high rate of positivity was identified in surgical specimens collected from necrotic tissue or abscesses in this study.^(20,21)^ The patients who did not have an etiologic diagnosis also did not have samples collected. Blood cultures, although less sensitive, may help to establish diagnoses in patients with septic shock or from whom a surgical sample was not obtained. The microbial results obtained from wound swabs may be less consistent, as non-pathogenic or colonizing microorganisms contaminating the superficial wound tissues may be detected, leading to inadequate antimicrobial therapy.

The rapidly changing epidemiology of infectious disease has placed MDR microorganisms as one of the most common SSTI pathogens, especially when healthcare-associated.^(5,11)^ In our study, we identified several patients infected with MDR bacteria, with the highest prevalence identified in cases of NF (1 in 4 patients from whom MDR bacteria were isolated), some of which were community-acquired and occurred in patients without traditional risk factors for antimicrobial resistance.

### Treatment

Management of complicated SSTIs often requires a combination of surgical debridement or drainage and empirical antibiotic therapy.^(5,17,20)^

While the selection of a surgical approach depends on the extent and location of the infection, the selection of antimicrobial therapy depends on the clinical presentation and etiology of the disease. In our study, empirical antimicrobial therapy was selected based on the type and site of the infection and presence of risk factors for MDR. The risk of mortality remained high in those who received appropriate empirical treatment and was highest in those who did not respond to empirical antibiotic treatment; however, this difference did not achieve statistical significance.

These data suggest that receipt of adequate antibiotic therapy is not the only determinant of mortality. We were unable to determine if the severity of the infection, extension of necrotic tissue, delays in antimicrobial therapy, presence of drug resistance, or presence of other infections/complications associated with the ICU accounted for the remaining variance in mortality. Although none of the patients in whom de-escalation was attempted died, switching to a narrower spectrum antibiotic should only be attempted when a good specimen from which antibiotic sensitive microorganisms can be isolated is available and patient condition is improving.

Every patient, except for those with cellulitis, required surgery on their first day in the ICU, some of whom required up to four procedures when a serial debridement approach was applied. Due to extensive debridement, vacuum-assisted wound closure therapy was successfully used in 2 (10%) patients with NF, and the other 2 (10%) patients were successfully treated with hyperbaric oxygen therapy also. These patients had more extensive disease and longer expected recovery times.

### Outcomes

Several clinical variables have been reported to be associated with mortality in NF.^(22)^ Our data showed that septic shock upon admission, history of cardiac arrest and leucocyte count were independently associated with mortality and, although not evaluated in our study, may also help in the diagnosis of NF.^(5,23)^ Infection severity upon admission, as indicated by SAPS II score, also predicted mortality, which we believe may reflect the multiorgan failure observed in NF.

Patients with NF had a high risk of mortality, even when appropriate treatment was received (aggressive fluid resuscitation, intravenous antibiotics, appropriate surgical debridement and supportive care), a result that was similar to that of other studies.^(11,24)^

The time interval between admission and the initial debridement has been described as the most important determinant of mortality in patients with NF, and delays of > 24 hours have been observed in association with increased mortality risk.^(25)^ Our data do not support these findings, a difference that was likely due to the sample size limitations, but do suggest that co-morbidities, nosocomial infections and ICU-related complications may also contribute to mortality.

The strengths of this study are that it revealed an SSTI epidemiology and etiology that was very different from that which has been traditionally described. NF, which was previously more common in trauma and war wounds, appears to be more strongly associated with iatrogenic skin infections and immunosuppression at present, and some patients appeared to develop severe infections in the absence of traditional risk factors. *S. aureus* was not the most commonly isolated microorganism. Additionally, while microorganisms with MDR were present even in the absence of traditional risk factors, prior use of antimicrobials was significantly associated with MDR isolation. Furthermore, we identified a high rate of positivity in surgical samples, which helped in establishing the etiology of infection. Although the identification of this finding was not the aim of this study, it may affect the manner in which clinicians select antimicrobials for the treatment of NF; however, whether it will affect patient outcomes remains unclear.

The limitations of this study are related to its retrospective nature. Some important data could not be retrieved, such as the timing of antibiotic initiation and details regarding the surgical approach and the extent of most infections. Additionally, as NF and complicated abscesses were rare conditions in the ICU, a limited number of patients with these conclusions could be assessed, precluding the performance of multivariate analyses or generation of survival curves for each type of infection. Therefore, questions regarding the influence of the timing of adequate empirical antibiotic therapy initiation, nosocomial infections and ICU complications on the risk of mortality remain to be answered.

## CONCLUSION

The crucial role of the recognition of life-threatening skin infections in facilitating early diagnosis, adequate surgical and medical treatment and intensive supportive care has been well-established. The rates of mortality and morbidity in necrotizing fasciitis have decreased slightly over the last decades; therefore, the recognition of risk factors and prognostic factors may help in the early diagnosis of necrotizing fasciitis and stratification of patient care. As risk factors change over time, so do causative microorganisms, and our study revealed a shift from traditional risk factors, such as trauma and wounds, to immunosuppression and surgical trauma and a shift from drug sensitive to drug-resistant bacteria. This raises questions regarding the selection of the best empirical antibiotic therapies. However, whether the selection of the best empirical antibiotic therapy will help reduce mortality is unknown, as mortality remained high even with adequate treatment, a finding that probably due to intensive care unit-associated complications and other infections. Efforts should be made to identify risk factors for skin and soft tissue infections in apparently healthy patients and targets to help in the early identification of infection and prevention of disease progression.
